# Lunar Regolith Geopolymer Concrete for In-Situ Construction of Lunar Bases: A Review

**DOI:** 10.3390/polym16111582

**Published:** 2024-06-03

**Authors:** Xiaowei Zheng, Cong Zhao, Xiaoyan Sun, Weiwei Dong

**Affiliations:** 1 College of Civil Engineering and Architecture, Zhejiang University, Hangzhou 310058, China; 22112081@zju.edu.cn (X.Z.); zhaocong5011@zju.edu.cn (C.Z.); 2 Shanxi-Zheda Institute of New Materials and Chemical Engineering, Taiyuan 030001, China; 3 Centre for Balance Architecture, Zhejiang University, Hangzhou 310063, China

**Keywords:** lunar regolith simulants, geopolymer, ISRU, mechanical property

## Abstract

The construction of lunar bases represents a fundamental challenge for deep space exploration, lunar research, and the exploitation of lunar resources. In-situ resource utilization (ISRU) technology constitutes a pivotal tool for constructing lunar bases. Using lunar regolith to create geopolymers as construction materials offers multiple advantages as an ISRU technique. This paper discusses the principle of geopolymer for lunar regolith, focusing on the reaction principle of geopolymer. It also analyzes the applicability of geopolymer under the effects of the lunar surface environment and the differences between the highland and mare lunar regolith. This paper summarizes the characteristics of existing lunar regolith simulants and the research on the mechanical properties of lunar regolith geopolymers using lunar regolith simulants. Highland lunar regolith samples contain approximately 36% amorphous substances, the content of silicon is approximately 28%, and the ratios of Si/Al and Si/Ca are approximately 1.5 and 2.6, respectively. They are more suitable as precursor materials for geopolymers than mare samples. The compressive strength of lunar regolith geopolymer is mainly in the range of 18~30 MPa. Sodium silicate is the most commonly utilized activator for lunar regolith geopolymers; alkalinity in the range of 7% to 10% and modulus in the range of 0.8 to 2.0 are suitable. A vacuum environment and multiple temperature cycles reduce the mechanical properties of geopolymers by 8% to 70%. Future research should be concentrated on the precision control of the lunar regolith’s chemical properties and the alkali activation efficacy of geopolymers in the lunar environment.

## 1. Introduction

**Figure 1:**
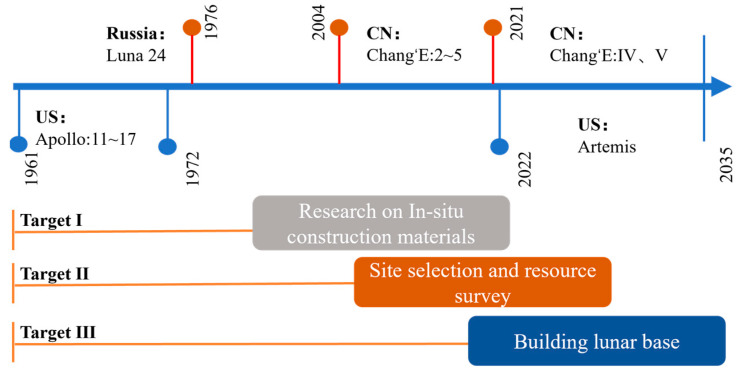
Overview of the international lunar explorations.

With the development of space technology in the 20th century, human understanding of the lunar environment has been constantly deepening [1]. Based on the remote sensing information obtained from the Apollo, Luna, and Chang’E lunar exploration mission series and the study of in-situ lunar regolith sampled from the lunar surface, it has been realized that the lunar environment contains rich resources. The course and goal of the lunar exploration project are shown in Figure 1 [2,3,4]. As space exploration expands in depth and breadth, the traditional space transportation methods originating from Earth will gradually show their limitations. In-situ resource utilization (ISRU) has become an essential technological tool that primarily includes extracting, transforming, storing, and utilizing material resources under specific lunar surface environment conditions, which runs through space activities and occupies an essential position in deep space exploration [5,6]. As the first station for deep space exploration, lunar bases are essential to exploiting lunar resources and conducting scientific research [6]. The challenges and difficulties of lunar base construction are the resistance to unfavorable environments, the exploitation and utilization of resources, and the highly automated requirements of the construction method [7]. Various materials have been developed for the in-situ construction of lunar bases, including sintering [8], fusion [9], bonding [10], and bio-polymerization [11]. Sintering and melting require significant energy input, and the molded specimens’ quality is not readily controllable. Cement, sulfur, and other binders cannot be produced on the lunar surface and must be obtained by rocket delivery. The alkali-activated lunar regolith geopolymer process addresses these shortcomings and has emerged as a technologically preferred option for ISRU [12,13].

Alkali-activated geopolymers are inorganic polymer materials formed by depolymerization and subsequent polymerization of silico-aluminate in an alkaline environment [14]. After being proposed in 1989, it has emerged as a prominent research topic in building materials because of its significant advantages of high strength, durability, and environmental friendliness [15,16,17]. The lunar regolith is widely distributed on the lunar surface and is a primary material source for in-situ construction in the lunar environment. To date, lunar sampling missions have retrieved hundreds of kilograms of lunar regolith from various regions and terrains of the lunar surface. Chemical analysis of these in-situ samples indicated that the lunar regolith is composed mainly of aluminosilicate materials, predominantly silicon, aluminum, and calcium, comprising over 80% of the material. Phase analysis indicates that between 10% and 36% of the aluminosilicate minerals in the lunar regolith are amorphous. These findings suggest that the lunar regolith shares properties similar to those of common geopolymer precursors [18,19,20]. Consequently, researchers have proposed using the alkali-activation process to produce lunar regolith geopolymer as a construction material [13,21]. The geopolymer reaction does not consume excessive water resources, as water can be extracted and recycled after the reaction [22]. As a result, this technology has an exceptionally high degree of in-situ resource utilization and low energy consumption [7].

Many factors influence the reaction products of alkali-activated geopolymers, including the type of precursor material, the specific alkaline activator used, its alkalinity, the reaction temperature, etc. Each of these elements significantly impacts the mechanical properties of the geopolymer [23,24,25]. Existing studies on alkali-activated geopolymers for lunar in-situ construction have utilized lunar regolith simulants. Variations in the production processes of these simulants have led to differences in their chemical properties, affecting the mechanical performance of the alkali-activated geopolymer made from lunar regolith simulants [21,26,27]. However, the existing studies lack uniform control parameters and processing procedures for lunar regolith simulants, resulting in disparate mechanical properties of geopolymers using lunar regolith simulants, which may impede the ability to accurately determine material parameters for the structural design of lunar bases. Additionally, more studies are needed on the effects of the lunar surface environment on geopolymers. This paper, therefore, aims to summarize and discuss the effect of the lunar surface environment on geopolymer concrete materials from the perspective of chemical reaction mechanisms. In order to standardize the processing flow of lunar regolith simulants, the differences in lunar regolith from different terrains were analyzed through a series of test data on the chemical and mineral composition of actual lunar regolith, and the key parameters affecting the chemical properties of lunar regolith were summarized. Subsequently, an approximate range of mechanical properties was obtained by synthesizing the research on the mechanical properties of alkaline-activated lunar regolith simulant geopolymers.

## 2. Geopolymer Materials

**Figure 2:**
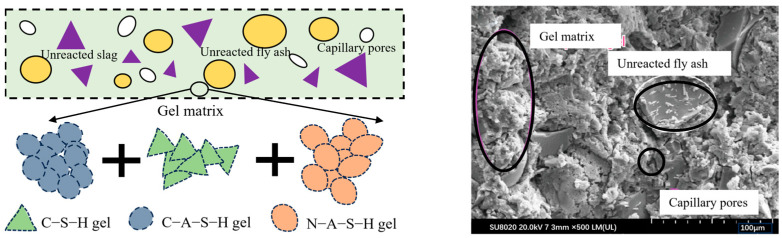
Composition of the geopolymer system [30].

Typical precursor materials for geopolymers on Earth encompass silico-aluminate materials, including fly ash (FA), metakaolin (MK), ground granulated blast furnace slag (GGBS), silica fume (SF), and volcanic ash (VA) [12,28,29]. Activators commonly used are substances that confer alkalinity to the cementitious system, such as sodium hydroxide (NaOH), potassium hydroxide (KOH), and sodium silicate (Na_2_SiO_3_) [12,25]. The geopolymer concrete is formed by solidification after mixing alkaline activators with precursor materials, and it can be divided into four scales from microscopic to macroscopic, which are disordered gel particles, gel matrix, slurry, and mortar/concrete. The gel matrix, mainly composed of hydrated calcium silicate (C-S-H), hydrated sodium silica-aluminate (N-A-S-H), and hydrated calcium silica-aluminate (C-A-S-H), plays a critical role in particle bonding, as illustrated in Figure 2.

### 2.1. Reaction Mechanism of Geopolymer

In the geopolymer precursors, the amorphous substances are the reactants involved in the alkali activation process. X-ray diffraction (XRD) experiments have shown that crystalline minerals have a long program with a tightly ordered atomic arrangement in equilibrium, and amorphous substances have a short program without long-range orders [31]. From a thermodynamic perspective, unlike crystalline minerals with a tightly ordered atomic structure, they maintain a metastable state and are in equilibrium [32]. For alkaline activation reactions, crystalline mineral forms of silico-aluminate minerals generally exhibit higher bond energies, whereas amorphous silico-aluminates feature weaker chemical bonds due to their internal structural disorder [33]. The reduced bond energy renders amorphous substances more reactive, making them more prone to reactions in an alkaline environment.

**Figure 3:**
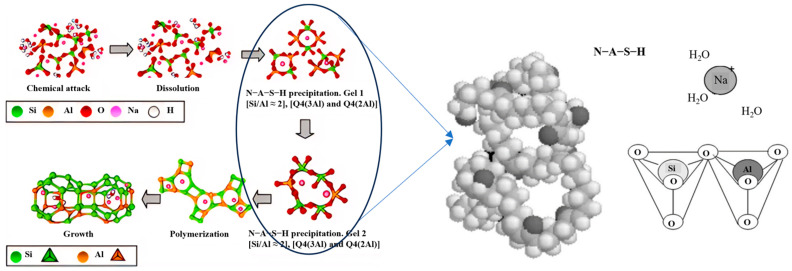
Reaction mechanism of geopolymer [15,30].

According to the chemical composition of geological polymer precursor materials, they are divided into three systems:

Low calcium system: Materials like VA and FA belong to this category. These materials contain less than 30% calcium and are dominated by silicon and aluminum. Due to the minimal presence of free calcium, the reaction products are mainly N-A-S-H, with a Young’s modulus of about 17 GPa [34,35]. Figure 3 shows the growth model for alkaline silica-aluminate gels proposed by Krivenko [30]. In this model, the N-A-S-H gel, an intermediate product, forms by substituting sodium ions and aluminum groups for silica tetrahedra. Initially, the ratio of Si/Al is close to 1 but approaches 2 in the later stages of the reaction. This shift occurs because Al-O bonds are more susceptible to breaking than Si-O bonds [36,37]. Duxson et al. [38] refined the model by confirming water’s distinct roles in the geopolymer reaction process compared to traditional cement materials. Water does not form the structure in the dehydration–condensation reaction process of geopolymers. Instead, water acts as a carrier in the alkaline-activation reaction. A two-phase structure of bound and free water is presented in the pores of the gels while the reaction is completed [39,40]. Most water can be extracted and recycled [22].

High calcium system: As GGBS, high-calcium FA, and other raw materials contain more than 30% calcium, the reaction rate is higher due to the higher calcium content in the system [25,41]. Calcium can sufficiently replace the sodium in the N-A-S-H, and the final product is primarily C-A-S-H. In C-A-S-H, calcium plays the cross-linking role, enhances the Si-O and Al-O skeleton bonding, and provides higher mechanical and durability properties than N-A-S-H. The Young’s modulus of C-A-S-H ranges from 13 to 48 GPa. When the Al/Si ratio in the system is around 0.4, the mechanical performance is superior. However, the shrinkage is significant and prone to dry cracking [42].

Mixture system: The low-calcium system has the disadvantages of a slow reaction rate and low mechanical properties. The high-calcium system has high mechanical properties and poor durability caused by high shrinkage cracking [43]. Therefore, a certain proportion of the mixture using GGBS, FA, and MK materials as precursor materials for geopolymer can compensate for the shortcomings of the above two systems; the reaction products contain N-A-S-H, C-S-H, C-A-S-H, and a small amount of mullite and hydrotalcite [44,45,46].

### 2.2. Factors Influencing Mechanical Properties 

Alkali-activated geopolymers were evaluated primarily based on their mechanical properties when used as construction materials. Many factors, such as the types of geopolymer raw materials and alkaline activators, significantly affect the mechanical properties of geopolymer concrete.

#### 2.2.1. Content of Amorphous Substances

**Figure 4:**
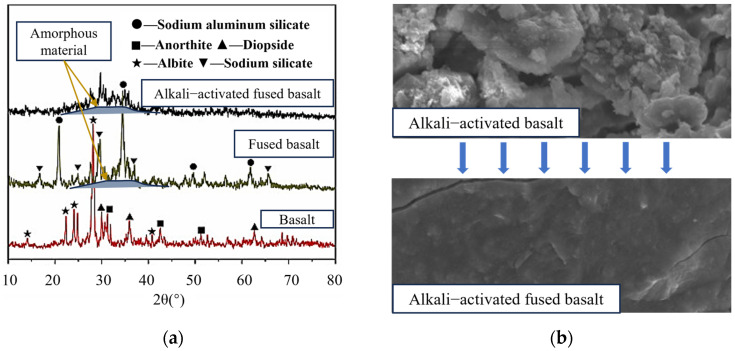
Comparison of alkali-activated basalt and fused basalt [51]: (**a**) XRD; (**b**) SEM.

Typically, the content of amorphous material in FA is between 30% and 60%, while GGBS contains more than 85% amorphous content [47,48,49]. In the XRD pattern, FA exhibits many sharp peaks, whereas GGBS exhibits a broad peak between 15° and 35° (2θ), with only a few weak sharp peaks. In comparable conditions, the compressive strength of FA geopolymer is typically 40% to 70% of that of GGBS geopolymer [29,50]. Mao et al. [51] demonstrated this by alkali-activating fine basalt powder containing only 4% amorphous content. The physical phase was transformed upon rapid cooling following calcination in a muffle furnace. A portion of the crystalline phase is transformed into amorphous, readily reactive substances. As shown in Figure 4a, the XRD pattern of untreated basalt exhibited many sharp peaks with maximal intensity, and the 7-day compressive strength was only 3.37 MPa when activated by Na_2_SiO_3_ solution. After melting treatment, the XRD pattern exhibited enhanced broad peaks between 25° and 35°. The 7-day compressive strength of the alkali-activated geopolymer reached 12.1 MPa. Figure 4b shows that the microstructure of alkali-activated basalt is a loose and porous arrangement with evident intergranular gaps. In contrast, the geopolymer microstructure of the alkali-activated melt-treated basalt exhibits a dense configuration, suggesting the generation of sufficient silico-aluminate gels to fulfill a bonding role.

#### 2.2.2. Chemical Composition

Due to the fixed chemical composition of single-component geopolymer precursors, especially the calcium content, controlling material properties can be challenging. For example, when GGBS is used as a precursor, its high calcium content (30% to 60%) leads to rapid reactions with alkaline activators, resulting in excessively short cure times and reduced workability [52]. Although such materials can achieve more than 75% of their final strength at 14 d, they are prone to significant shrinkage or cracking over time [47]. Conversely, using only FA, which belongs to a low calcium system, results in a slower rate of alkaline activation, leading to inadequate setting and curing or poor mechanical properties. FA geopolymer can only develop 30% to 60% of its mechanical properties at 14 d [48]. Consequently, most existing geopolymer precursor materials employ composite components in appropriate proportions, which regulate the reaction rate and products to achieve the desired effect on mechanical properties and durability [41,53]. The existing studies of alkali-activated geopolymers are shown in Table 1.

Further research has shown that adding an appropriate quantity of solid particles with a certain degree of inertia to the reaction system provides nucleation points for gel precipitation, which is conducive to the cross-linking of the gel and improves the anti-chlorine ion penetration properties of geopolymers [54,55]. These materials are typically blended with GGBS, and their dosage must be carefully controlled to avoid adverse effects on mechanical properties. Lou et al. [56] found that with the substitution rate increasing to 10%, the average compressive strength of 28 d was decreased by 9.6%, and the chloride diffusion coefficient was reduced by 46.5%. In addition, Huang et al. [54] used 20% quartz powder instead of GGBS to increase the mechanical strength of the geopolymer by 12%. Moreover, the particle size and specific surface area of the precursor materials also influence the extent of the reaction. Materials with a larger specific surface area have a greater contact area with the activator in the system, forming more gel and providing higher mechanical properties [57]. The mechanical properties of FA with a median particle size of 7.87 μm are 90% higher than those of geopolymer with a median particle size of 38.5 μm [58].

**Table 1:** Three systems of alkali-activated geopolymer.

System	Precursors	Activator	n(SiO_2_/Na_2_O)	Na_2_O%	Compressive Strength (MPa)	Ref.
High calcium	GGBS	Na_2_SiO_3_	2.6	15%	44.9~78.4	[53]
	GGBS	Na_2_SiO_3_	2.6	12%	41.0~75.4	
	GGBS	Na_2_SiO_3_	1.5~2.4	14%	47.3~76.2	[29]
Low calcium	FA	Na_2_SiO_3_	1.2	8%	27.4~41.7	[28]
	FA	Na_2_SiO_3_	0.5~1.0	4%	19.6~42.3	[59]
	FA	Na_2_SiO_3_	1.0	8%	23.0~64.0	
	FA	Na_2_SiO_3_	0.6~1.5	7%	24.9~43.6	[48]
	FA	Na_2_SiO_3_	2.0	9%	24.3~32.0	[60]
	FA	KOH+ Na_2_SiO_3_	2.2	9%	22.0~26.0	
Mixed	GGBS+ FA	Na_2_SiO_3_	0.5~2.3	12%	30.0~56.0	[61]
	VA	Na_2_SiO_3_	1.5	14%	33.2	[62]
	VA	NaOH	2.5	6%	22.0	[50]

#### 2.2.3. Activators

The type and dosage of activator are critical factors in the performance of geopolymer concrete. Alkalinity is measured as the mass fraction of Na_2_O in the cementitious material. Higher alkalinity promotes the dissolution of cementitious materials, increasing the number of silico-aluminate groups involved in the reaction and producing more gel products that contribute to mechanical properties. However, when the alkalinity exceeds a certain threshold, the mechanical properties of the geopolymer begin to deteriorate. The most commonly used alkalinity ranges from 4% to 12% [63]. 

**Figure 5:**
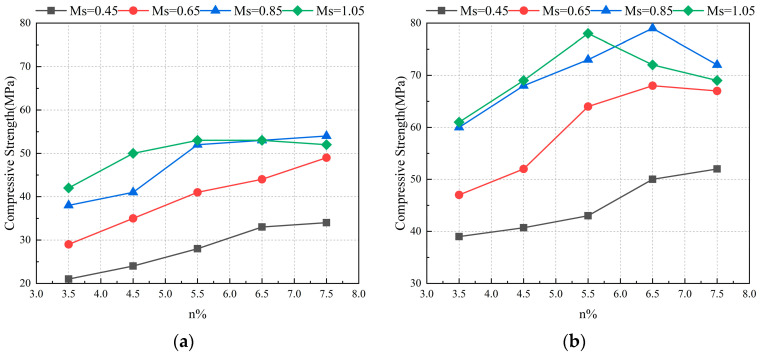
Compressive strength of the GGBS geopolymer based on modulus and alkalinity [64]: (**a**) 7 days; (**b**) 90 days.

Na_2_SiO_3_ provides alkalinity and introduces additional SiO_2_, which affects the ratio of Si/Al in the system due to the modulus (n(SiO_2_/Na_2_O)) in Na_2_SiO_3_. Taghvayi et al. [64] explored the effect of different doping amounts of Na_2_SiO_3_ on mechanical properties when used as an activator. As shown in Figure 5, the temporal compressive strength at two ages, 7 d and 90 d, exhibited disparate patterns. During the curing period of up to 7 d, the compressive strength increased with alkalinity and modulus. When the modulus exceeded 0.85, the impact of elevating the alkalinity on the compressive strength became less pronounced. Furthermore, when the modulus reached 1.05, the strength of the geopolymer was no longer elevated following the elevation of the alkalinity to 5.5%. Upon curing the geopolymer specimens for 90 days, it became evident that increasing the alkalinity and modulus had a more pronounced effect on the mechanical properties. However, when the modulus exceeded 0.85 and the alkalinity exceeded 5.5%, further increments in the alkalinity resulted in a decline in compressive strength. Consequently, during the geopolymer reaction process, the alkalinity and modulus in the system exert a mutual influence, and an optimal modulus and alkalinity exist to obtain the highest mechanical properties [64,65].

In conclusion, incorporating higher amorphous content will form more silica-aluminum groups, which serve as reaction materials. Among these, the reactivity of the calcium element is the most pronounced, exhibiting the capacity to effectively enhance the reaction rate and degree of reaction while simultaneously improving the mechanical properties of geopolymers. Using sodium silicate as an activator facilitates the geopolymer reaction by regulating the elemental percentage within the reaction system. The optimal range for alkalinity and modulus control is associated with the highest mechanical properties.

### 2.3. Applicability of Geopolymers in the Lunar Environment

The lunar environment exhibits notable differences from that of the Earth. It is characterized by extreme temperature variations, vacuum conditions, cosmic radiation, moonquakes, and low gravity, which can affect the reaction process, rheological properties in the freshly mixed state, and hardened mechanical properties. Furthermore, the high levels of cosmic radiation and moonquake disturbances present significant challenges for geopolymer materials’ radiation and impact resistance. Consequently, it is paramount to assess the suitability of geopolymers as construction materials in the lunar environment.

#### 2.3.1. Temperature

Temperature significantly impacts the reaction process of geopolymers, with the early curing temperature directly influencing the final mechanical properties. The most substantial improvement in the mechanical properties of geopolymers occurs when the curing temperature is between 40 °C and 85 °C [28,66]. When the curing temperature is 60 °C, the mechanical properties are enhanced to 32% relative to those at 25 °C. However, when the temperature exceeds this threshold, the enhancement rate of the mechanical properties begins to decline [28]. In environments up to 500 °C, the residual strength of geopolymer remains above 85% of that with 25 °C as the environmental temperature [67,68,69]. When the temperature exceeds 500 °C, the stiffness of the geopolymer begins to decrease, and sintering gradually occurs [70]. Additionally, it can withstand low temperatures and has a specific resistance to the impact of temperature change. Xiong et al. [21] placed the geopolymer in 0 °C and −30 °C curing for 7 d, respectively. The compressive strength of the specimen remained above 50 MPa, and the residual strength was 90% after undergoing the action of temperature cycling 40 times in the temperature range of −196 °C to 25 °C. Zhang et al. [26] found that the flexural strength of geopolymer decreased by 49% to 70% and the compressive strength decreased by 15% to 18% when subjected to mid-latitude temperature cycling on one lunar day.

Table 2 summarizes the temperature conditions observed on the lunar surface, ranging from −233 °C to 140 °C. The temperature difference between lunar daylight and sunset is significant, and the lunar day and night cycle is long, with one lunar day equaling 28 earth days [3]. Previous studies indicate that geopolymers can adapt to the extreme temperatures of the lunar surface. It is also important to note that the geopolymer reaction requires liquid water as a carrier. When the temperature is below 0 °C, it is impossible to guarantee that the activator will be liquid. Therefore, it is advisable to consider performing the work when the temperature exceeds 0 °C or artificially heat the area to maintain the proper temperature. A temperature cycle with a large amplitude can weaken their performance significantly. Therefore, environmental temperatures should be considered when selecting lunar base sites.

#### 2.3.2. Moonquake

The moonquake monitors deployed by Apollo have monitored approximately 500 seismic activities occurring on the lunar surface each year. The interval between each occurrence is approximately 0.7 earth days, with 81% of these events classified as deep-source seismic, with a relatively small magnitude equivalent to a Richter earthquake of 2~3 [71,72]. Therefore, the moonquake does not directly damage the building structures in a low-gravity environment but negatively impacts the structural performance during construction. The seismic frequency is typically concentrated between 0.3 and 12 Hz, with a duration of approximately 30 to 120 min [73]. Many factors influence the setting process of geopolymer, including the type of precursor material, the environmental conditions, the alkali activators, the temperatures, etc. The solidification time of FA geopolymer was found to be 2.6 h at the initial stage and 21 h at the final stage. However, when the precursor material was added to 35% GGBS, the initial and final setting times were adjusted to 40 min and 80 min, respectively. Furthermore, the initial and final setting times are shorter when the maintenance temperature increases to 60 °C [74]. By adjusting the ratio of Si/Al in the precursor material to a value below 1.1, the final setting time of the address polymer could be controlled within 400 min [75]. This process allows the completion of setting to occur during the moonquake interval, which is beneficial for resisting the seismic shock.

Freshly mixed geopolymer belongs to the Bingham fluid category. It has been demonstrated that vibration significantly affects the rheological properties of the fluid [76]. The vibration frequency and duration are sensitive technical parameters. By subjecting the fresh mortar to vibrations at approximately 25 Hz, the geopolymer and aggregate undergo relative motion, resulting in a slump decrease of 4.7% to 7.7% and an increase of 25% to 33% in 2 h slump loss over time [77,78,79]. The utilization of lunar geopolymer concrete as a raw material and the construction thereof via the 3D printing concrete process represent the prevailing direction of ISRU [80]. The perturbation caused by the moonquake will specifically affect the geopolymer in its fresh state, thereby affecting the construction and building processes; it must be considered in material design and structural analysis. However, at the present stage, more concrete material performance tests and analyses are lacking for the impact of moonquakes. Furthermore, the influence mechanism must be clarified, as it restricts the development of in-situ additive manufacturing of lunar regolith concrete technology and the implementation of intelligent construction of lunar base programs.

#### 2.3.3. Vacuum

The lunar surface’s atmosphere is thin and creates a vacuum environment [81]. The compressive strength of the geopolymer exhibited a reduction of 8% to 15% following conditioning in a vacuum environment. This phenomenon can be attributed to the vacuum’s capacity to accelerate the volatilization of water within the system, thereby increasing the porosity of the geopolymer after molding [22,82]. Curing geopolymers in a vacuum environment at 25 °C has a detrimental effect on their mechanical properties after 28 days. The residual strength rate is approximately 60% and is elevated to 80% when the curing process occurs at 80 °C or above. This phenomenon, known as the early high-temperature effect, accelerates the polymerization reaction, allowing the geopolymer to react sufficiently before the moisture is lost, which reduces the number of pores [83]. However, the existing environments are simulated by vacuum curing chambers with a vacuum level of about 2 × 10^−6^ Pa. The lunar surface vacuum level varies between 10^−11^ Pa and 10^−14^ Pa, and the effect of higher vacuum levels on the mechanical properties of geopolymers has not yet been investigated [84].

#### 2.3.4. Radiation

**Figure 6:**
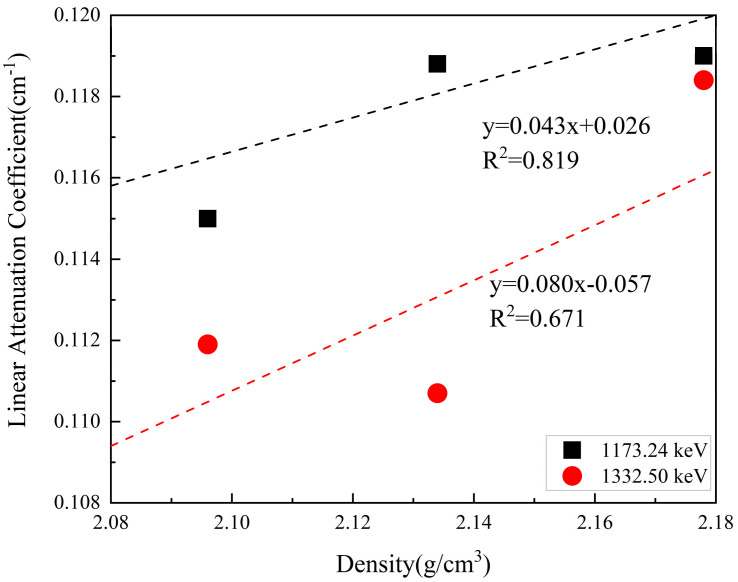
The radiation attenuation coefficient of FA geopolymer [24].

The lunar surface has intense cosmic radiation, with an annual dosage of approximately 300 mSv. The maximum permissible radiation dosage for workers in the radiation area is 50 mSv per year. Furthermore, cosmic radiation can affect instruments’ precision [85]. Therefore, the lunar base must provide sufficient shielding against radiation to guarantee the normal conduct of scientific research and the health of researchers. Concrete is a commonly used radiation-shielding material for nuclear projects. Its higher density provides a high level of radiation isolation, and a high density of lead is usually mixed as aggregates [86,87]. Studies have shown that geopolymers with FA and GGBS as precursor materials possess good radiation resistance, and the specimen’s density shows a linear trend with the radiation attenuation coefficient, as shown in Figure 6. The growth rate of the radiation attenuation coefficient with increasing density is 0.043. A linear attenuation coefficient of 0.12 can be achieved for a 2.1 g/cm^3^ material density. As the radiation intensity increases from 1173.24 keV to 1332.50 keV, the growth rate of the radiation attenuation coefficient with increasing material density increases to 0.08 [24].

The density of the lunar regolith samples ranges from 1.2 to 3.2 g/cm^3^. The formation of geopolymers in the lunar regolith after alkali activation results in higher densities [2,88]. Consequently, the Fuller grading curve is optimized based on the continuous grading theory to achieve the maximum mixed material density [89]. This approach can maximize radiation shielding performance. A significant quantity of lunar regolith can be utilized to cover the surface of a building; thereby, radiation levels are reduced by more than 50% when the thickness of the cover exceeds 1 m [90]. Consequently, alkali-activated lunar regolith polymers can serve as protective building materials against radiation.

Table 3 summarizes the characteristics of each environmental factor and the challenges faced when geopolymers are applied to the lunar surface environment.

In conclusion, silico-aluminate mineral precursor materials with suitable chemical element composition ratios and high amorphous content can be alkaline-activated to produce geopolymers with excellent mechanical properties. Geopolymers are well suited for the lunar surface environment when used as construction materials. Consequently, lunar regolith, which also comprises silico-aluminate minerals, has the potential to serve as precursor materials for geopolymers. However, it is still necessary to enhance the reliability of geopolymers for lunar regolith by conducting tests on their applicability for extreme temperature variation, high vacuum, and moonquakes within the lunar surface environment.

## 3. Lunar Regolith as a Precursor

**Figure 7:**
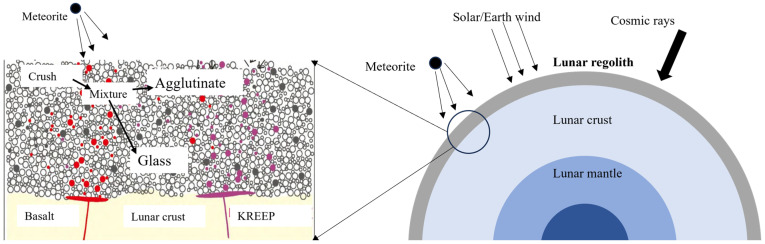
Structure of the lunar and environmental conditions of the regolith.

Figure 7 shows the lunar structure and external environment. The lunar regolith is the fine-grained part that covers the surface at a depth of 3 to 20 m [91]. It is readily available and abundant and has become a preferred source of in-situ building materials.

The lunar atmosphere is thin, with a density far less than one hundred trillionth of the earth’s atmosphere [92]. Due to the lack of atmospheric protection, meteorites and minor asteroids can impact the lunar surface, with impact speeds reaching 20 to 40 m per second. The lunar magnetic field’s strength is less than one-tenth of that of the earth. Additionally, the depth to which solar wind and cosmic rays penetrate the lunar surface can reach ten meters [93]. Consequently, the lunar regolith, in addition to undergoing the formation of volcanic eruptions, also undergoes a unique weathering process [94].

The series of lunar regolith samples collected from equatorial, mid-latitude regions and the northern Lumke Mountains by Apollo, Luna, and Chang’e covers different formation periods and landforms [95]. According to the different sampling locations, lunar regolith samples can be classified into three categories: mare, highland, and mare–highland. There are some notable differences in the chemical and mineralogical characteristics of different lunar regolith samples [84].

### 3.1. Chemical Composition of the Lunar Regolith

The lunar regolith is rich in silica-aluminate, with a SiO_2_ mass fraction accounting for 40~50%. Additionally, it contains a range of chemical components, including Al_2_O_3_, MgO, CaO, Ti_2_O, and FeO. The ratios of SiO_2_, Al_2_O_3_, and CaO in the precursor materials can significantly affect the reaction process of alkali-activated geopolymers. Therefore, it is necessary to analyze the differences in the Si/Al and Si/Ca ratios of different samples and select suitable samples to use as precursor materials.

**Figure 8:**
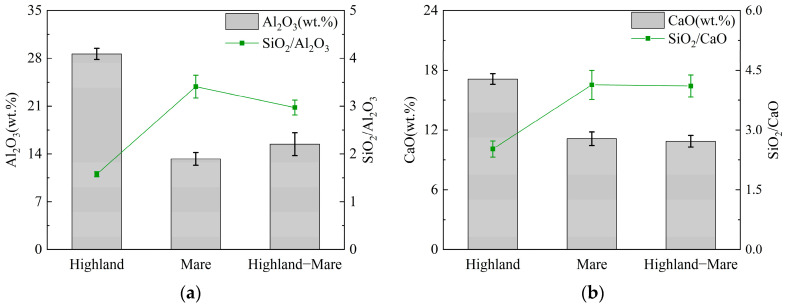
Differences in elemental composition of lunar regolith samples from different regions: (**a**) Al_2_O_3_ content and Si/Al ratio; (**b**) CaO content and Si/Ca ratio.

Figure 8 shows the relative proportions of the three principal elements in each category of lunar regolith samples. The Apollo 16 samples from the highland exhibit a markedly higher proportion of aluminum by mass, approximately 28%, with an average Si/Al ratio of 1.5. Mare samples exhibit the lowest proportion of aluminum, approximately 12%, with a Si/Al ratio of 3.4 [3,18]. Combining two topographic layers, samples from the mare–highland junction have a higher aluminum content than those from the mare samples (Figure 8a) [96]. Similarly, the highest elemental calcium content was found in the highland samples, at approximately 17%, with a Si/Ca ratio of 2.6. In contrast, the mare samples exhibited only 10% elemental calcium and a high Si/Ca ratio of 4.0 (Figure 8b).

Geopolymer precursors with an appropriate chemical element ratio interval exhibit higher mechanical properties. For silica-aluminum-rich precursors, such as MK, with an aluminum percentage of 35% or more and a Si/Al ratio of approximately 1.2, the alkali-activated geopolymer tends to possess higher mechanical properties later [23,97]. For precursor materials rich in calcium–silicon, such as GGBS, the Si/Al ratio ranges from 1.2 to 3.0, and the Si/Ca ratio ranges from 0.4 to 0.9. The high calcium content will result in the geopolymer possessing high early strength. In general, precursor materials with Si/Al ratios of 2 and below exhibit higher volcanic ash activity [96]. The highland samples’ Si/Al and Si/Ca ratios meet the requirements, rendering them more suitable as geopolymer precursor materials regarding chemical element compositions than the mare samples.

### 3.2. Mineral Composition of the Lunar Regolith

**Figure 9:**
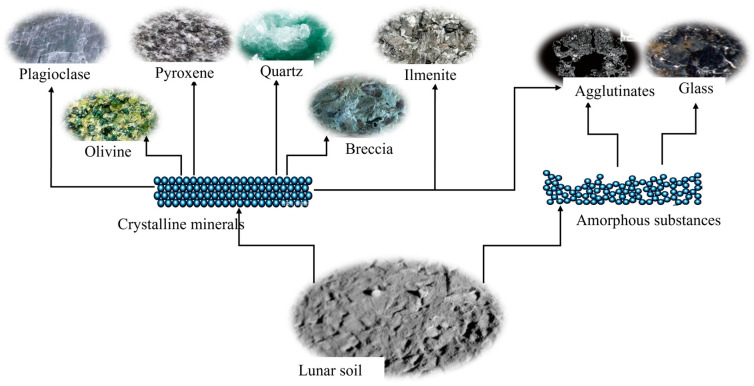
Mineralogical composition of the lunar regolith.

Figure 9 shows the mineral composition of the lunar regolith, primarily composed of glass, plagioclase, pyroxene, and olivine, with a minor component of ilmenite [98]. The genesis of lunar regolith amorphous substances can be attributed to two factors. Firstly, during periods of lunar volcanism, fine particles formed by volcanic eruptions are transformed into a glassy material called volcanic glass due to the rapid cooling rate. Secondly, the thin lunar atmosphere and the decisive impact of meteorites triggered explosions, with the resulting impact and high-temperature effects leading to the fragmentation and molten vaporization of the lunar basalt, followed by rapid cooling, replenishing some of the glass. The lunar regolith underwent a vitrification process, forming glass as agglutinates, slag-like substances formed by glass-bonded rock particles [99].

**Figure 10:**
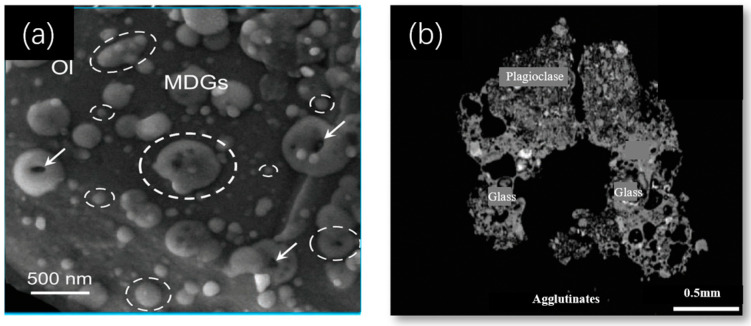
Amorphous substances in lunar regolith: (**a**) glass [101]; (**b**) agglutinate [2].

**Figure 11:**
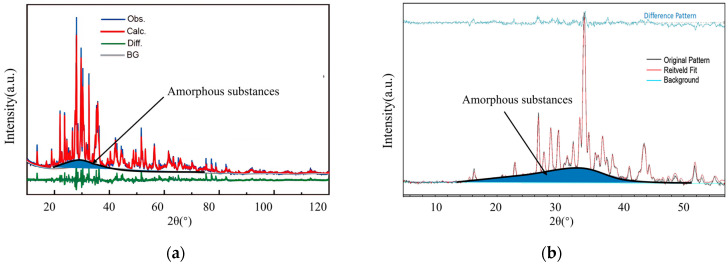
XRD pattern of a lunar regolith sample: (**a**) CE5C044 [102]; (**b**) Apollo 69661 [103].

A large amount of glass at the scale of micrometers to sub-micrometers is present on the surface of the lunar regolith particles, and the amorphous substances consist of a combination of separate glass (Figure 10a) and agglutinate (Figure 10b) [100]. The amorphous substances appear as broad bun peaks in the XRD pattern. Figure 11 shows the XRD pattern of CE5C044 belonging to the mare samples and Apollo69661 belonging to the highland samples, while the broad peaks at 20°~80° (2θ) represent the lunar regolith amorphous substances [2].

The composition of the lunar regolith is influenced by genesis and environmental factors, resulting in a slight variation in the percentage of different mineral types. Table 4 presents the statistical data on the percentage of significant minerals in the typical lunar regolith samples. The rate of amorphous substances in the highland lunar regolith is higher than that in the mare lunar regolith. Furthermore, the content of amorphous substances in the Apollo 12, Apollo 16, and Luna 20 series belonging to the highland samples is significantly higher than that of the mare samples, which is 31% to 36% [3,104]. Given that amorphous substances serve as the primary precursor material in the geopolymer reaction, a higher content of amorphous substances allows for a more thorough alkali activation, resulting in the production of more significant quantities of silico-aluminate gel, which plays a more critical role in bonding, ultimately leading to enhanced mechanical properties. Regarding amorphous content, highland lunar regolith samples are more suitable as precursors for developing alkali-activated lunar regolith geopolymer.

**Table 4:** Proportional mineral composition of the lunar regolith.

Specimens	Amorphous Substances (wt.%)		Crystalline Minerals (wt.%)			Ref.
	Glass	Agglutinate	Plagioclase	Pyroxene	Olivine	
CE-5	15.5		30.4	44.5	3.6	[2]
Apollo (67461)	15.4		72.2	8.7	4.8	[104]
Apollo (64501)	32.0		45.2	5.2	14.1	
Apollo (69961)	35.3		51.8	5.3	5.6	
Apollo (12001)	32.4		19.2	34.9	7.5	
Apollo (12044)	31.9		17.7	16.9	5.7	
Apollo (15531)	27.6		16.0	42.7	4.1	
Apollo (15558)	14.5	7.3	14.2	42.2	1.0	
Apollo (10018)	7.7	13.5	—	—	—	
Apollo (10094)	4.8	5.5	—	—	—	
Luna16	15.1	32.3	16.1	15.8	5.7	[105]
Luna20	8.2	26.6	25.3	13.2	3.2	[106]

### 3.3. Reaction Mechanism of Alkali-Activated Lunar Regolith Geopolymer

Researchers have found that lunar regolith can be activated to manufacture geopolymer concrete with considerable strength in alkaline environments [13,27,107].

**Figure 12:**
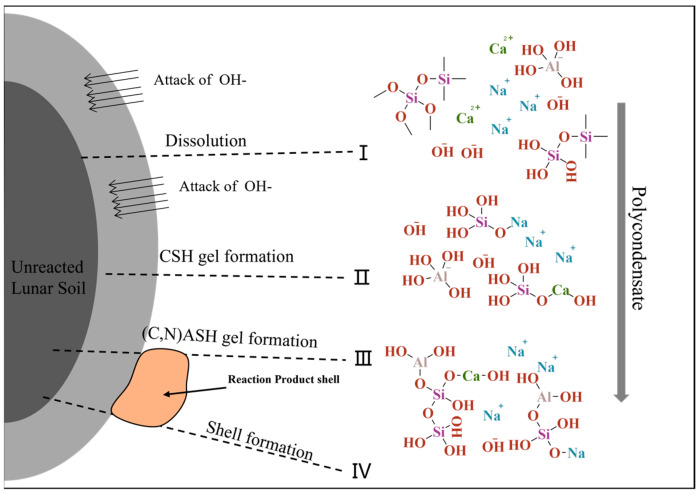
Reaction mechanism of the lunar regolith-based geopolymer system.

The highland lunar regolith sample could be a geopolymer precursor material. The amorphous substance in the highland lunar regolith contains approximately 15% elemental calcium and 35% elemental aluminum, with a Si/Al ratio of 1.5 and a Si/Ca ratio of 2.6, characteristic of the alkali-activated mixed system. The alkali-activated reaction process can be divided into four steps: depolymerization, condensation, crystalline hardening, and precipitation into shells. Based on the chemical composition of lunar regolith, the reaction process of alkaline-activated lunar regolith can be inferred, as shown in Figure 12.

The reaction process can be divided into four stages:

1. Depolymerization: Induced by the action of OH^−^, it results in the dissolution of amorphous lunar regolith constituents, including CaO, into a free ionic state. This process involves the disruption of Si-O-Si, Si-O-Al, and Al-O-Al bonds, which hydroxyl groups subsequently replace. This process results in the formation of various free radicals.

CaO + H_2_O → Ca^2+^ + 2OH^−^

SiO_2_ + H_2_O + OH^−^ → SiO(OH)_3_^−^

Al_2_O_3_ + 3H_2_O + 2OH^−^ → 2Al(OH)_4_^−^

2. Condensation: As the degree of depolymerization increases, the number of groups in the system rises, and the contact between the groups intensifies, leading to an increase in the polycondensation degree, with Na^+^ and Ca^2+^ in the system replacing some of the hydroxyl groups. This results in the formation of a condensation gel, which occurs concurrently with the formation of a gel in the system containing C-S-H.

SiO(OH)_3_^−^ + Na^+^ ↔ SiO(OH)_3_Na

SiO(OH)_3_Na + Ca^2+^ +OH^−^ ↔ SiO(OH)_4_Ca + Na^+^

3. Al^3^⁺ addition: Al^3^⁺ dissolution is slower, and in the third stage, Al(OH)₄^−^ groups are replaced, forming Si-O-Al bonds once again. The product at this stage is (N,C)-A-S-H.

SiO(OH)_3_^−^ + Al(OH)_4_^−^ → (OH)_3_AlOSiO(OH)_2_^2−^ + H_2_O

SiO(OH)_2_^−^ + Al(OH)_4_^−^ → (OH)_3_AlOSiO_2_(OH)_2_^3−^ + H_2_O

4. Shell formation: The gel product continues to be generated and precipitates into a shell until the precipitation hardens.

In conclusion, the samples of lunar regolith materials retrieved from different landforms exhibit some differences in chemical element composition and amorphous content. In conjunction with the alkali-activated geopolymer reaction principle, the highland lunar regolith, which contains a higher amorphous material content, exhibits higher chemical reactivity. The silicon, aluminum, and calcium ratio within this type can facilitate the formation of C-A-S-H, which possesses superior mechanical properties.

## 4. Research on Alkali-Activated Lunar Regolith Geopolymer

### 4.1. Lunar Regolith Simulants

Due to the limited availability of lunar regolith samples, researchers have developed multiple versions of lunar regolith simulants to assess the viability of producing alkali-activated lunar regolith polymers. These simulants are crafted based on lunar regolith samples’ physical and chemical properties. However, the differing objectives behind the development of each simulant can result in variations in specific properties. A summary of the existing simulated lunar regolith is provided in Table 5. Lunar regolith simulants can be classified into two types based on raw materials and production processes.

Type I: Designed to simulate physical properties derived from minerals such as basalt, MLS-1, and JLU-H. These materials simulate the chemical composition and geotechnical properties of the lunar regolith but do not account for the amorphous substances within the minerals.

Type II: Designed to simulate chemical and physical properties. Examples of this type include materials such as JSC-1, JSC-1A, and CAS-1. This type employs volcanic ash, which has a formation process analogous to lunar regolith and includes a certain number of amorphous substances.

**Table 5:** Development and research on lunar regolith simulants.

Category	Name	Year	Agency	Source	Feature	Ref.
I	JLU-H	2022	Jilin University	Damiao Mine, Chengde City, Hebei Province	Focus on the simulation of the lunar regolith crystalline phase	[108]
	ISRM-1	2023	Chinese Academy of Sciences	VA, titanium magnetite	Focus on the simulation of the Apollo 17 sample’s titanium	[109]
	TJ-1	2011	Tongji University	Volcanic ash from Jingyu County, Jilin Province	Focus on geotechnical properties.	[110]
	MLS-1	1990	University of Minnesota	Basalt	Does not contain amorphous substances, with a smaller average particle size	[111]
II	JSC-1	1993	Johnson Space Center	Volcanic ash from Merriam Crater, Arizona	Non-residual	[112]
	CAS-1	2009	Chinese Academy of Sciences	Volcanic ash from Jingyu County, Jilin Province	The median grain size is chemically similar to that of the Apollo 14 lunar regolith sample	[113]
	JSC-1A	2010	Johnson Space Center	Volcanic ash from Merriam Crater, Arizona	A substitute for JSC-1	[114]
	BP-1	2013	Geological Survey Denver	Black Point lava flow, Arizona.	The grain size is more akin to the lunar regolith than JSC-1	[115]
	BH-1	2020	Beijing University of Aeronautics and Astronautics	Volcanic ash from Huinan County, Jilin Province	Particle size distribution close to the Apollo 17 lunar regolith sample	[83]
	DNA-1	-	ESA	Italy	Amorphous substance simulations were considered	[82]
	LHS-1	2021	University of Central Florida	Mineral and rock debris	Similar to the highland sample	[116]
	LMS-1	2021	University of Central Florida	Mineral and rock debris	Similar to the mare sample	

To approximately determine the methods of manufacturing geopolymer using lunar regolith simulants, it is of the utmost importance to select a type of lunar regolith simulant that closely mimics the properties of the actual lunar regolith. This entails ensuring similarities in the content of amorphous substances, chemical composition, particle size, specific surface area, particle morphology, etc. Most alkali activation experiments on lunar regolith employed the second type of lunar regolith simulants.

### 4.2. Mechanical Properties of Alkali-Activated Lunar Regolith Simulants

Table 6 presents a synthesis of existing research on geopolymers using lunar regolith simulants. The precursor materials are the Type II lunar regolith simulants, which are physically and chemically similar to lunar regolith samples. Alkali activators such as NaOH, Na_2_SiO_3_, and K_2_SiO_3_ were utilized. Significant variability is evident in the 28-day compressive strength reported in various studies.

Wang et al. [22] adopted VA as a lunar regolith simulant and attained a compressive strength of 50.36 MPa with an 8% alkalinity of NaOH. The compressive strength of the geopolymer using JSC-2A was found to be 54.85 MPa, while that of LN was 59.60 MPa [21,117]. The median particle size of JSC-2A is 35.3 μm, while the median particle sizes of JSC-1A, OPRL2N, OPRH2N, and EAC-1A are 68.2 μm, 58.0 μm, 46.2 μm, and 181.0 μm, respectively. Additionally, the amorphous content of JSC-2A is 51.0%, considerably higher than that of the authentic lunar regolith sample. The mechanical properties of the geopolymer utilized as the precursor material for JSC-2A are so elevated as to be unrepresentative. The reactivity of these three lunar regolith simulants differs significantly from other simulants due to the degree of control over the content of amorphous substances and their chemical composition. The 28-day compressive strength ranged from 12.25 MPa to 31.86 MPa for the remaining lunar regolith simulants. The identical quantity of activator was utilized to activate different lunar regolith simulants, and it was discovered that, except for JSC-2A, the 28-day compressive strength of geopolymer mainly ranged from 19.20 MPa to 24.35 MPa, which means that these lunar regolith simulants exhibit similar reactivity during the alkali activation [117]. BH-1 is a simulant of the mare sample with 16% aluminum content, and the addition of metakaolin and alumina reagents can increase the aluminum content of the precursor material. The addition of 5% metakaolin resulted in a 71% increase in compressive strength and a 28% increase in flexural strength, thereby enhancing the mechanical properties of the lunar regolith geopolymer [83].

Nevertheless, incorporating an additional aluminum source also entails elevated transportation costs for lunar construction scenarios due to the augmented necessity for Earth-to-lunar transportation. Different types and dosages of activators were used to activate two lunar regolith simulants, LHS-1 and LMS-1 [118]. The results demonstrated that the mechanical performance when using Na_2_SiO_3_ as the activator was significantly higher than when using K_2_SiO_3_. Highland samples can generate a greater quantity of silico-aluminate gel following alkali activation, which results in markedly superior mechanical properties in the highland simulant LHS-1 compared to the mare simulant LMS-1. The mare lunar regolith samples contain higher MgO to form N-(M)-S-H gel with porous and low mechanical properties [119,120]. This corroborates the analysis presented in Section 3.1, which indicates that highland samples are more suitable as precursor materials for geopolymers. As the concentration of the activator increases, so does the alkalinity in the system, which further promotes the formation of geopolymers.

**Table 6:** Mechanical properties of alkali-activated lunar regolith simulants.

Precursor	w/b	Activator	Na_2_O%/ K_2_O%	n(SiO_2_/Na_2_O)	28-Day Compressive Strength (MPa)	Temperature (°C)	Ref.
BH-1	0.28	NaOH	9%	—	13.00~32.00	40~80 °C	[80]
VA	0.27	NaOH	~8%	—	32.46~50.36	−196~25 °C	[22]
LHS-1	0.25	K_2_SiO_3_	~7%	0.8	12.25	76~105 °C	[118]
LHS-1	0.25	K_2_SiO_3_	~8%	1.0	16.73		
LMS-1	0.25	K_2_SiO_3_	~7%	0.8	13.87		
LMS-1	0.25	K_2_SiO_3_	~8%	1.0	18.09		
LHS-1	0.23	Na_2_SiO_3_	~7%	0.6	27.95		
LHS-1	0.23	Na_2_SiO_3_	~8%	0.8	41.23		
LMS-1	0.23	Na_2_SiO_3_	~7%	0.6	24.59		
LMS-1	0.23	Na_2_SiO_3_	~8%	0.8	31.86		
JSC-1A	0.21	Na_2_SiO_3_	~8%	2.0	24.35	60 °C	[117]
JSC-2A					54.85		
OPRL2N					22.47		
OPRH2N					22.96		
EAC-1A					19.20		
LN	0.26	Na_2_SiO_3_	8%	1.4	59.60	60 °C	[21]

**Figure 13:**
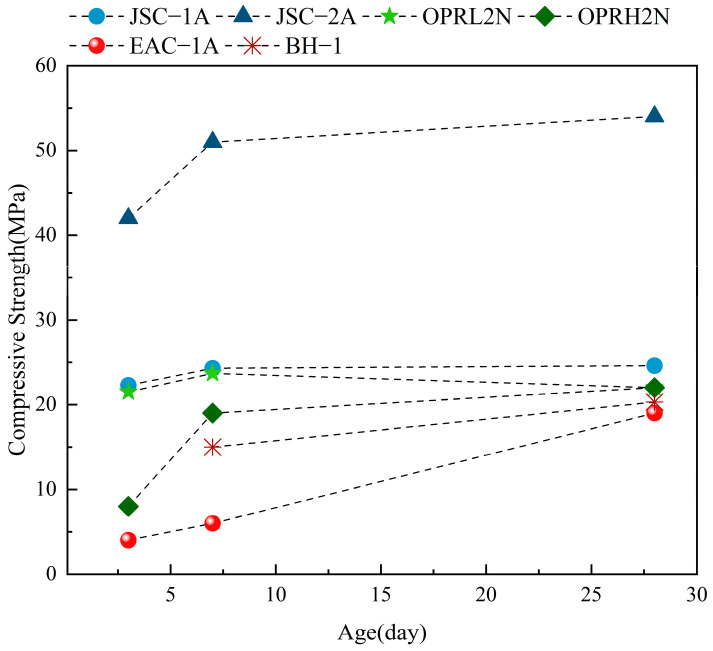
Compressive strength development patterns of alkali-activated lunar regolith simulants.

In order to investigate the strength development pattern of alkali-activated lunar regolith simulants, the experimental compressive strength data at different ages in Table 6 were tabulated in Figure 13. The mechanical properties in the early stages appear to have different development patterns. A higher amorphous content promotes the development of the reaction. The majority strength of JSC-1A and OPRL2N, with amorphous contents of 49.9% and 45.4%, respectively, has been developed at 7d. In contrast, EAC-1A mechanical properties grow linearly. Only 32% of the compressive strength is developed at 7 d. Moreover, the 28-day compressive strength of the EAC-1A geopolymer is also smaller than the rest of the group, suggesting that the lunar regolith with a lower amorphous content has a relatively low reactivity [83,117].

### 4.3. Advantages of Lunar Regolith Geopolymer

Table 7 presents a synopsis of extant research on construction materials for in-situ construction of lunar bases. Many manufacturing processes have been proposed to develop building materials using lunar regolith, such as sintering, casting, hot pressing, bonding, and biopolymer [8,121,122,123]. Among them, the processes of sintering, casting, and hot pressing require a significant amount of energy to support [124]. Traditional bonding and hot-pressing processes require a large amount of cement and sulfur as binders, resulting in low in-situ utilization and posing challenges to space transportation technology. The process of bio-polymerization has the advantages of low energy consumption and the disadvantages of low product strength, making it difficult to withstand loads [7].

The lunar regolith with a particle size of 5~75 μm can be used as geopolymer precursor materials, and the lunar basalt debris with a particle size of 0.1~5 mm can be used as aggregates. The lunar ice, with a reserve reaching 0.1~3 billion tons, can be cyclically used as water for in-situ construction [132]. Only a small amount of solid alkaline activators must be transported to complete the fabrication of alkaline-activated lunar regolith geopolymers in the lunar environment. By adjusting the alkali-activated lunar regolith geopolymer to suitable rheological properties before setting, the contour extrusion 3D printing concrete process could be adopted to construct the lunar base and pavement with high automation, high precision, a high degree of freedom, and high material savings [80,133]. This new bonding process dramatically reduces transportation between the Earth and the lunar surface. It has significant technical advantages in improving the utilization of in-situ resources and reducing energy consumption.

In conclusion, the existing lunar regolith simulants lack conformity in their amorphous content. Additionally, there are significant discrepancies in the particle size distribution, which results in notable differences in the mechanical properties of alkali-activated geopolymers produced from different simulants. Therefore, it is essential to control the simulation control parameters for the lunar regolith simulants and the fabrication process. Compared to other construction processes, the geopolymers produced by the alkaline-activated process exhibit advantageous characteristics, including low energy consumption and high strength, which indicate feasibility and applicability as in-situ lunar building materials.

## 5. Future Perspectives

Previous research has demonstrated the feasibility of alkali-activated lunar regolith concrete and its numerous advantages in scenarios involving the construction of the lunar surface. Nevertheless, there are still several aspects that require further investigation.

1. At the current stage, retrieving lunar regolith samples on a large scale for testing purposes is not feasible. Consequently, lunar regolith simulants remain the most available material for testing purposes. There is still a lack of a unified standard for preparing lunar regolith simulants. The simulated materials are designed to replicate the chemical properties of lunar regolith and primarily utilize volcanic ash, with variations in amorphous substances, chemical compositions, and particle size distributions derived from different volcanic sources.
2. The type and quantity of alkaline activators exert a considerable influence on the mechanical properties of the final lunar regolith geopolymer. Given that activators for in-situ construction of lunar bases must be transported by rocket, selecting the most appropriate type of activator is essential to achieving optimal activation efficiency.
3. Vacuum and temperature variations have adverse effects on geopolymers. Therefore, future research needs to consider the degeneration of geopolymer solidification under the high vacuum and extreme temperature conditions of the lunar surface environment. The accurate and quantitative performance evaluation of geopolymer is essential to designing safe and reliable architectural structures fabricated with lunar regolith polymer.

## 6. Conclusions

Using lunar regolith for alkali activation to produce geopolymers is a highly feasible process within the context of lunar ISRU technology schemes. This process offers multiple advantages over other methods.

1. The lunar regolith geopolymer reaction mechanisms belong to the mixed system, and the types of gels generated are mainly N-A-S-H and C-A-S-H. The chemical and mineralogical compositions of the highland and the mare lunar regolith exhibit notable differences. Highland lunar regolith samples contain approximately 35% amorphous substances; the ratio of Si/Al is approximately 1.5; the content of aluminum is approximately 28%; the ratio of Si/Ca is approximately 2.6; and the content of calcium is approximately 17%. Compared with the mare lunar regolith, the highland lunar regolith is more suitable as a geopolymer precursor material.
2. Previous studies on lunar regolith simulants can be divided into two types. The first type focuses on the simulation of physical properties, while the second type focuses on the simulation of physical and chemical properties. The lunar regolith simulants for alkali-activated geopolymers belong to the second type. The mechanical properties of alkali-activated lunar regolith simulants are mainly concentrated in the range of 18 MPa to 30 MPa, except for test data exhibiting significant differences in amorphous content compared to the lunar regolith samples. Sodium silicate is the most commonly used activator for lunar regolith geopolymers, and alkalinity in the range of 7% to 10% and modulus in the range of 0.8 to 2.0 are suitable.
3. Geopolymers can be adaptable for lunar surface construction environments, and the residual compressive strength after multiple temperature cycles within the temperature range of −40 °C to 120 °C, simulated in the lunar surface environment of the mid-latitude region, is above 70%. The vacuum environment on the lunar surface will prematurely evaporate the water inside the reaction system, increasing the porosity of the geopolymers. A vacuum degree of approximately 2 × 10^−6^ can decrease the mechanical properties of the geopolymer by 8~40%.
4. The interval between deep-source moonquakes is approximately 16.8 h. The final setting time of the geopolymer can be adjusted to less than 400 min. Therefore, the curing process can be completed within the interval between moonquakes. The duration of the deep-source moonquake is approximately 30 to 120 min, which may influence the rheological properties of the freshly mixed geopolymer. Therefore, vibration disturbance must be considered when designing materials and structures for in-situ lunar construction.

## Figures and Tables

**Table 2:** Temperature variations in different lunar regions (°C).

	Polar Shadow Crater	Remaining Polar Regions	Equator	Mid-Latitudes
Average temperature	−233	−53	−19	−53~−19
Range	—	10	140	110

**Table 3:** Challenges posed by the lunar surface environment.

Factors	Features	Challenges
Temperature	Range: −230~140 °C	Low temperatures impede the chemical reaction. Temperature-induced cyclic shock has a detrimental effect on the mechanical properties of geopolymers.
Moonquake	Frequency: 500 occurrences per year, equivalent to an earthquake of magnitude 2 on the Richter scale Duration: 30~120 min	Frequent seismic activities affect the rheological properties of the fresh-state geopolymers and the required setting time.
Vacuum	Vacuum level: 10^−11^~10^−14^ Pa	A vacuum can prematurely evaporate the water in the reaction system, increasing the geopolymer’s internal porosity and weakening its mechanical properties.
Radiation	Radiation dose: 300 mSv per year	Geopolymers need to be as dense as possible, and sufficient weathering should be applied to cover the building surface so that it absorbs the radiation.

**Table 7:** Processing techniques for lunar regolith building materials.

Precursor	Earth material	Technique	Temperature (°C)	Compressive Strength (MPa)	Ref.
JSC-1A	—	Melting	1070~1125	152	[125]
DNA	—			213	
CAS-1	AlSi10Mg powder	Melting (Laser)	1700	264	[123]
CAS-1	—	Melting	1200~1500	1012~1955	[126]
FJS-1	—	Sintering	200~600	33	[127]
JSC-1A	—	Sintering (Laser)	50~200	4	[128]
JSC-1A	Al	Sintering (Reduction-oxidation reaction)	660	10~18	[129]
JSC-1	Mg		100	10.2	[130]
——	Portland cement	Hot pressing (Steam autoclave)	175~203	75	[10]
JSC-1	Sulfur		193	31~33	[131]
CAS-1	Portland cement	Hot pressing (Steam autoclave)	190	27	[107]
JSC, OPR	Alkali activator	Reaction solidification (Geopolymer)	80	25~54	[117]
BH-1	Alkali activator		37~90	4~21	[80]
LN	Alkali activator		25~80	29~46	[22]
JSC-1A	Protein matrix	Reaction solidification (Biopolymer)	25	4~13	[11]
LHS-1	Protein matrix		40	20~40	[122]

## Data Availability

Not applicable.
